# Assessing the accuracy of substance use disorder treatment search tools: A cross-sectional analysis of national and state-level directories

**DOI:** 10.1016/j.dadr.2024.100249

**Published:** 2024-06-25

**Authors:** Sean R. Riley, Leslie P.M. Brouwer, Daniel E. Jonas

**Affiliations:** a Division of General Internal Medicine, Department of Internal Medicine, The Ohio State University College of Medicine, Columbus, OH, United States; b Division of Health Services Management and Policy, The Ohio State University College of Public Health, Columbus, OH, United States

**Keywords:** Alcohol & drug use, Treatment assessment and planning, Service delivery systems, Referrals, Public policy issues

## Abstract

**Background:**

Addressing the critical public health crisis of substance use disorder (SUD), this study evaluates the accuracy of SUD treatment search tools, such as FindTreatment.gov, to connect patients with appropriate care.

**Methods:**

To ensure geographic diversity, we randomly selected one state from four distinct US regions (Arizona, Florida, Massachusetts, Ohio) and then randomly selected counties of varying sizes (one large, two medium, three small) within each state using a random number generator. Contact information for practices was extracted from the tools and validated through phone calls. The primary outcome measures were exact accuracy rate (wherein all information was accurate) and functional accuracy rate (wherein enough information was provided to facilitate care establishment).

**Results:**

A total of 697 practices from within ten SUD treatment search tools were assessed. Accuracy of the ten SUD treatment search tools varied considerably, with exact accuracy rates ranging from 9.1 % to 76.0 % (mean: 56.0 %) and functional accuracy rates from 50.0 % to 92.0 % (mean: 82.8 %). National tools exhibited higher accuracy for both exact accuracy rate (66.3 % v. 49.0 %; p = 0.2864) and functional accuracy rate (83.8 % v. 82.2 %; p = 0.9148) than state tools, while privately funded tools demonstrated higher accuracy for both exact accuracy rates (66.8 % v. 48.9 %; p = 0.2008) and functional accuracy rates (83.8 % v. 82.2 %; p = 0.9148), but none of these differences were statistically significant.

**Conclusions:**

This study found that SUD treatment search tools commonly list inaccurate information, underscoring the need for systematic improvements in data management and validation practices.

## Highlights

• Access barriers to treatment exacerbate the substance use disorder crisis.
• Substance use disorder search tools and directories have low to moderate accuracy.
• National tools are more accurate than state-specific tools.
• Privately funded tools are more accurate than publicly funded tools.

## 1 Introduction

The substantial morbidity and mortality from substance use disorder represents a critical public health crisis, exacerbated by the proliferation of synthetic opioids such as fentanyl (Substance Abuse and Mental Health Services Administration (SAMHSA), 2023b). According to the Centers for Disease Control and Prevention (CDC), 2021 and 2022 were the first two years in US history with over 100,000 deaths attributable to substance overdose (Ahmad et al., 2023). Many evidence-based treatment options exist (Substance Abuse and Mental Health Services Administration (SAMHSA), 2023c), including inpatient detoxification and rehabilitation (de Andrade et al., 2019), group or individual counseling (Dellazizzo et al., 2023), and pharmacotherapy (McPheeters et al., 2023), yet access barriers remain.(Priester et al., 2016) One such barrier is challenge locating adequate treatment options (Balio et al., 2022). Despite recent uptake of screening, brief intervention, and referral to treatment (SBIRT) in clinical settings (Nunes et al., 2017), healthcare providers have reported not knowing where to refer patients, largely due to the complexities of navigating different levels of care needed by patients for different substances or challenges with health insurance coverage (Blevins et al., 2018). These challenges are further compounded by additional access barriers, such as inability to afford care, patient hesitance due to stigma, or simply the lack of treatment options in a given location (Bunn et al., 2019). To help fill in knowledge gaps and better connect patients with care, federal, state, and non-profit entities have created a variety of substance use disorder treatment search tools and directories.

Web-based search tools and directories are managed by governmental or non-profit organizations on the national or state-level to facilitate the identification of and referral to substance use disorder treatment. Table 1 provides an overview of national and state tools, including description of the organization’s role and the types of information they provide. To address the question of whether these search tools fulfill their mission to deliver reliable and accessible information to patients, this study aimed to assess the accuracy of various national and state-based search tools operated by either governmental or non-profit organizations.

**Table 1:** Overview of substance use disorder treatment search tools and directories.

**Tool**	**Managing Organization**	**Scope**	**Inclusion Criteria**	**Type of Information Provided**
FindTreatment.Gov (Substance Abuse and Mental Health Services Administration (SAMHSA), 2023a)	Federal agency; publicly funded	National	Accredited treatment facilities and providers	• Services Offered (e.g. inpatient, outpatient) • Substances Treated (e.g. alcohol) • Accepted Insurances (e.g. Medicaid) • Financial Aid Options (e.g. sliding scale) • Special Populations (e.g. LGBTQ, veterans) • Languages Supported (e.g. Spanish) • Transport Options (e.g. nearest bus stop) • Facility Type (e.g. private, public) • Licensure (e.g. accreditation)
Commission on Accreditation of Rehabilitation Facilities (CARF)’s Find a Provider tool (Commission on Accreditation of Rehabilitation Facilities, 2023)	Non-profit accreditation agency; privately funded	National	CARF-accredited facilities and providers	• Services Offered (e.g. inpatient, outpatient) • Special Populations (e.g. LGBTQ, veterans) • Licensure (e.g. accreditation)
National Association of Addiction Treatment Providers (NAATP)’s Addiction Industry Directory (National Association of Addiction Treatment Providers, 2023)	Non-Profit professional society; privately funded	National	Member treatment providers	• Services Offered (e.g. inpatient, outpatient) • Financial Aid Options (e.g. sliding scale) • Special Populations (e.g. LGBTQ, veterans) • Languages Supported (e.g. Spanish) • Facility Type (e.g. private, public) • Licensure (e.g. accreditation)
Start Your Recovery’s Find Support tool (Start Your Recovery, 2023)	Non-profit recovery organization; privately funded	National	Various treatment facilities and support services across the US	• Services Offered (e.g. inpatient, outpatient) • Substances Treated (e.g. alcohol) • Accepted Insurances (e.g. Medicaid) • Financial Aid Options (e.g. sliding scale) • Special Populations (e.g. LGBTQ, veterans) • Languages Supported (e.g. Spanish) • Licensure (e.g. accreditation)
Bureau Substance Addiction Services Licensing System (Bureau of Substance Addiction Services, 2023)	State agency, Massachusetts; publicly funded	State	Facilities and providers licensed to provide addiction care in Massachusetts	• Services Offered (e.g. inpatient, outpatient) • Licensure (e.g. accreditation)
HelplineMA’s Find Help tool (The Massachusetts Substance Use HELPLINE, 2023)	State agency, Massachusetts; publicly funded	State	Various treatment facilities and support services in Massachusetts	• Services Offered (e.g. inpatient, outpatient) • Accepted Insurances (e.g. Medicaid) • Financial Aid Options (e.g. sliding scale) • Special Populations (e.g. LGBTQ, veterans)
Relink.org (Relink, 2023)	Non-profit organization, Ohio; privately funded	State	Various treatment facilities and support services in Ohio; emphasis on faith-based care	• Services Offered (e.g. inpatient, outpatient) • Hours of Operation • Accepted Insurances (e.g. Medicaid) • Facility Type (e.g. private, public) • Licensure (e.g. accreditation)
Arizona Health Care Cost Containment System (AHCCCS) Opioid Treatment Locator (Arizona Health Care Cost Containment System, 2023)	State agency, Arizona; publicly funded	State	Opioid treatment programs certified by SAMHSA	• Services Offered (e.g. inpatient, outpatient) • Hours of Operation • Bed Availability/Accepting New Patients • Accepted Insurances (e.g. Medicaid) • Special Populations (e.g. LGBTQ, veterans) • Languages Supported (e.g. Spanish)
Florida Department of Children and Families (DCF)’s Find Local Services tool (Families, 2023)	State agency, Florida; publicly funded	State	Verified treatment facilities and providers	• Contact Information only
Florida Behavioral Health Association (FBHA) (Florida Behavioral Health Association, 2023)	Trade organization, Florida; privately funded	State	Member treatment providers	• Contact Information only

## 2 Methods

We utilized a stratified random sampling method using a random number generation in Excel, focusing on counties within four states, each within different geographic regions of the US (Northeast, Midwest, South, and West). Within each of the four selected states (Arizona, Florida, Massachusetts, Ohio), we used a random number generator in excel to randomly select one large county (population greater than 500,000), two medium counties (population between 100,000 and 500,000), and three small counties (population less than 100,000). County population data was sourced from the U.S. Census (United States Census Bureau, 2024).

We evaluated the accuracy of contact information for all practices listed within each county on the tools listed in Table 1. The national tools included in this study were selected based on their comprehensive service coverage, ensuring they provide resources to the entire nation. For state-level tools, we sampled various states and selected tools that serve the entire state. We included tools that the authors had pre-existing knowledge from previous work in substance use disorder research. The discovery of additional tools was conducted via Google searches, focusing on those that met our criteria for broad accessibility and coverage. Two reviewers extracted phone numbers, addresses, and website URLs for all included practices. The reviewers compared results, discussed discrepancies, and reached consensus on any differences in their assessment. The address and phone number for each practice was then validated for these data points via phone call, while the website was validated by searching the websites online. Validation occurred between September 2023 and December 2023.

The primary outcome measures were exact accuracy rate (wherein all contact information was accurate) and functional accuracy rate (wherein enough information was provided to facilitate care establishment).

If a practice did not answer phone calls after three attempts (each call made at least one week apart, on different days of the week, at different times of the day), then they were classified as "Unable to Validate". If the researcher was placed on hold for more than 5 min, it was also classified as “Unable to Validate”. If a practice stated that they do not offer substance use treatment, it was classified as “Inaccurate”, even if the contact information provided was accurate. In order for practice contact information to be classified as “functionally accurate”, one of the following situations had to take place: the website or address was inaccurate, but the phone number was accurate and connected you to a live person who could provide correct address and website; the phone number was inaccurate, but the website listed a correct phone number that connected you to a live person; or the phone number connected you to a different practice within the same health system or health network, and you could be directed to the initial practice.

After evaluating the accuracy of contact information, given the lack of normality in our dataset, we conducted Mann-Whitney U tests to compare the exact and functional accuracy rates between national and state tools as well as publicly funded and privately funded tools. We hypothesized that national-level substance use disorder treatment search tools would demonstrate higher exact and functional accuracy rates compared to state-level tools and that privately funded tools would exhibit higher accuracy rates than those funded by public sources. These hypotheses were generated through our team’s clinical and personal experience with these search tools. STATA 17.0 was used to statistically assess differences in the accuracy of search tools across these dimensions (StataCorp, 2023). This research was exempt from IRB review.

## 3 Results

Table 2 provides the accuracy rates for all included search tools as well as averages across different scopes (e.g. national or state) and organization type (e.g. publicly funded or privately funded). At least one state-specific search tool from each state was included in the analysis. A total of 697 practices were assessed across all tools. Across all states, 46 practices had information that was unable to be validated. National tools ranged considerably in the number of practices included in our sample: Substance Abuse and Mental Health Services Administration (SAMHSA)’s FindTreatment.gov provided 395 practices assessed in our sample, while the National Association of Addiction Treatment Providers (NAATP) provided 25 practices. National-level tools assessed an average of 243 practices, and they covered all counties across the sampled states. State-level tools ranged from 144 practices assessed to seven practices assessed. State-level tools, operating within individual states, assessed an average of 68 practices.

**Table 2:** Accuracy rates for various search tools.

	Number of Practices Assessed	Exact Accuracy Rate (%)	Functional Accuracy Rate (%)
Search Tools (raw)			
FindTreatment.Gov	395	65.3	79.2
CARF^a^	242	61.6	83.5
NAATP^b^	25	68.0	88.0
StartYourRecovery.org	313	70.3	84.4
Bureau SAS^c^ (MA)	120	63.3	88.3
HelplineMA^d^ (MA)	101	36.6	75.3
Relink^e^ (OH)	144	67.4	79.2
AHCCCS^f^ (AZ)	25	76.0	92.0
DCF^g^ (FL)	11	9.1	72.7
FBHA^h^ (FL)	7	42.9	85.7
Overall (mean)	138	56.0	82.8
National	243	66.3	83.8
State	68	49.2	82.2
Publicly funded	110	48.9	82.2
Privately funded	181	66.8	83.8

^a^ Commission on Accreditation of Rehabilitation Facilities.

^b^ National Association of Addiction Treatment Providers.

^c^ Bureau of Substance Addiction Services; servicing Barnstable, Dukes, Franklin, Hampden, Nantucket and Norfolk counties.

^d^ Massachusetts Substance Use Helpline; servicing Barnstable, Dukes, Franklin, Hampden, Nantucket and Norfolk counties.

^e^ Relink.org; servicing Erie, Mahoning, Montgomery, Pike, Union, and Wood counties.

^f^ Arizona Health Care Cost Containment System; servicing Apache, Gila, La Paz, Pima, Pinal, and Yuma counties.

^g^ Florida Department of Children and Families; servicing Gulf, Levy, Santa Rosa, Washington, Saint Lucie, and Miami-Dade counties.

^h^ Florida Behavioral Health Association; servicing Gulf, Levy, Santa Rosa, Washington, Saint Lucie, and Miami-Dade counties.

Across all tools assessed, there was notable variation in the exact accuracy rates of all tools, ranging from 9.1 % to 76.0 %. The average exact accuracy rate across all tools was 56.0 %. Functional accuracy rates varied from 50.0 % to 92.0 %, with an average rate of 82.8 %. For the comparison on scope, national tools exhibited higher accuracy for both exact accuracy rate (66.3 % v. 49.0 %; p = 0.2864) and functional accuracy rate (83.8 % v. 82.2 %; p = 0.9148) than state tools, but the differences were not statistically significant. Among the national tools assessed, StartYourRecovery.org had the highest exact accuracy rate (70.3 %), while NAATP had the highest functional accuracy rate (88.0 %). On the state level, the Arizona Health Care Cost Containment System’s Treatment Locator had both the highest exact and functional accuracy rates (76.0 % and 92.0 %, respectively).

For comparison on organizational type, privately funded tools demonstrated higher accuracy for both exact accuracy rates (66.8 % v. 48.9 %; p = 0.2008) and functional accuracy rates (83.8 % v. 82.2 %; p = 0.9148), but the differences were not statistically significant. Among the privately funded tools assessed, StartYourRecovery.org had the highest exact accuracy rate (70.3 %), while NAATP had the highest functional accuracy rate (88.0 %). Of the publicly funded tools, the Arizona Health Care Cost Containment System’s Treatment Locator had both the highest exact and functional accuracy rates (76.0 % and 92.0 %, respectively).

## 4 Discussion

This study found considerable variation in both exact and functional accuracy rates across substance use treatment search tools, with overall accuracy observed to be low to moderate for many tools. National tools demonstrated slightly higher accuracy rates compared to state-level tools, and privately funded tools outperformed government-funded tools. The relatively low accuracy of some tools underscores the challenges faced by healthcare clinicians and patients in effectively navigating treatment options for substance use disorders.

Search tools are one of many potential steps on the pathway to connecting patients to adequate treatment. Their integration with other healthcare services, such as referrals from primary care clinicians or mental health professionals, is essential in forming a cohesive treatment network. However, common barriers, such as stigma, geographical constraints, and financial limitations often hinder access to treatment. In this context, the accuracy of such tools becomes vital in ensuring patients are connected to treatment as reliable information can help with overcoming such access barriers.

The observed low accuracy rates may be attributable to several factors. First, healthcare services, and substance use treatment services in particular, are subject to considerable churn as law, policy, and health demands shift. Additionally, treatment centers may frequently change their services, contact information, and operations. This is compounded by the decentralized nature of US healthcare with a mixture of private and public providers and varying state-level regulations. Second, there might be a gap in collaboration and communication between the entities managing these tools and the treatment providers. For example, national tools may be relying on reporting from state-based entities, which leads to delays and discrepancies in data reporting. Third, funding for certain tools may lapse, leading to a cessation of regular updates. Lastly, there may be insufficient verification processes in place to ensure that the data initially entered was itself accurate.

Our findings support our hypotheses that national and privately funded tools would perform better than state-level and publicly funded tools, respectively. While these differences were not statistically significant, there still may be various reasons for the observed discrepancy in reported accuracy. National tools likely benefit from better financing and a wider scope of service, allowing for more experience and expertise to generate over time. Private tools may have access to more flexible funding streams, enabling them to invest in advanced technology and more frequent data updates. In contrast, public tools may face bureaucratic constraints and relatively limited budgets, which can hinder their ability to update information promptly and invest in technology. This study highlights the critical need for systematic improvements to substance use disorder treatment search tools. Governments and non-profit organizations that host such tools should invest in improving data management strategies to ensure practice information is up-to-date and reliable, including integrating machine learning. Another strategy would be to increase collaboration between the practices and hosting organizations, which has ancillary benefits by fostering stronger partnerships that can help with education and further patient enrichment. Policies should consider building the necessary infrastructure and appropriately financing such endeavors, including incentives to organizations for accurate data. We need to better understand the role these tools play in patients’ pathway to care. Insight from other countries may prove useful. Future research should focus on changes in tool accuracy over time, the efficacy of different data management strategies, and the impact of these tools on patient outcomes, including access to and quality of care as well as substance poisonings.This study had several limitations. First, we did not assess whether tools included real-time availability of access to appointments or vacancies, which are essential for effective locator tools to facilitate timely patient care. Most organizations appear to lack the resources for such updates. Only one tool included in our sample (relink.org) offers treatment slot availability information, but our analysis did not evaluate the accuracy of that particular feature of the tool. Others tools in states not included in our study may also track treatment slot availability and investigating the accuracy of those tools would be a value-added contribution to future research. Second the sample was confined to a limited number of counties in a limited number of states. This may limit generalizability of our findings, but we believe the random sample selection of different size counties in states in different regions of the country strengthened our external validity. Third, not all search tools have the same mission, so the number of practices assessed varied considerably between the tools. Some tools only included practices accredited by their organization, while others were broader. Fourth, the cross-sectional design of this study means that corrections or updates may have been made to some of the tools between when we collected our data and when we validated the data by phone call. Lastly, there may have been some discrepancies arising from classification of phone calls that resulted in extended periods of being put on hold (greater than five minutes); these were classified as “unable to validate”.

## 5 Conclusion

This study demonstrates that the accuracy of substance use disorder treatment search tools is generally low to moderate, with national and privately-funded tools slightly outperforming state-level and publicly funded ones, respectively. These findings underscore the need for improved data management and validation processes in these tools to enhance their reliability and effectiveness in aiding treatment access.

## Funding

The authors have no funding to declare.

## CRediT authorship contribution statement

**Sean Riley:** Conceptualization, Methodology, Software, Validation, Formal analysis, Investigation, Writing – original draft; **Leslie Brouwer:** Software, Validation, Investigation, Writing – review & editing; **Daniel Jonas:** Resources, Writing – review & editing, Supervision

## Declaration of Competing Interest

The authors have no financial/personal interest or belief that could affect their objectivity.
